# The Presence of Alchohol in Animal Tissues

**Published:** 1880-05

**Authors:** 


THE PRESENCE OF ALCOHOL IN ANIMAL TISSUES.

To clear up some points hanging on the investigations of Schrader and Dresch, the following experiments were tried by J. Bechamp. (Compt. rend. 1879, lxxxix 573.) A piece of horse flesh, weighing three kilog., was dipped in boiling water for ten minutes, and placed in a dish on the 8th of June, and closely covered up with a thick linen cloth. On the 16th of July, the flesh had become very foul and full of life, but the air does not appear to have penetrated to the centre. Alcohol, amounting to 0.8 gramme was obtained, a part of which was burnt, the rest oxidized with chromic acid to aldehyde, and then to acetic acid, the soda salt of which was prepared and identified. In addition to this, about ten grammes of sodium acetate, butyrate and salts of other higher acids were obtained. Another mass of horse flesh, weighing four kilog. was simply left to itself for four days, and a subsequent treatment yielded alcohol as before, but in less quantity, as well as acetic and butyric acids. The next question which suggested itself to the author, was to determine whether alcohol forms a constituent of a living organ. It has been shown by A. Bechamp, that alcohol is a normal constituent of urine and milk ; the question, hence, arises whether it also occurs in the tissues. Fresh sheep's liver, immediately after the animal was killed, contained alcohol ; fresh and still warm sheep's brains also contained it, and in larger quantity than the liver ; fresh and still warm bullock's brains also contained it. These results show that the presence of alcohol in the tissues does not necessarily indicate poisoning.—*Popular Science Review*, January 1880.

